# Effects of Harvesting Periods and Cultivar on the Physicochemical and Sensory Properties of Two Coffee Bean Varieties

**DOI:** 10.3390/foods14173135

**Published:** 2025-09-08

**Authors:** Guanru Huang, Shuaimin Liu, Gan-Lin Chen, Yuan Zhao, Qiulan Huang, Qingjing Cen, Er-Fang Ren

**Affiliations:** 1 Guangxi Subtropical Crops Research Institute, Guangxi Academy of Agricultural Sciences, Nanning 530001, China; huangguanru@gxaas.net (G.H.); liushuaimin1992@163.com (S.L.); ganlin-chen@163.com (G.-L.C.); 15277134380@163.com (Y.Z.); huangqiulan@gxaas.net (Q.H.); 13065100103@163.com (Q.C.); 2 Key Laboratory of Quality and Safety Control for Subtropical Fruit and Vegetable, Ministry of Agriculture and Rural Affairs, Nanning 530001, China; 3 Guangxi Key Laboratory of Quality and Safety Control for Subtropical Fruits, Nanning 530001, China

**Keywords:** coffee, harvest periods, cultivated varieties, physicochemical property

## Abstract

Coffee (*Coffea* sp.) bean variety and harvesting periods are factors that directly affect its overall quality. In this study, we investigated the effects of four different harvesting periods (December, January, February and March) on the physicochemical and sensory properties of two coffee bean Catimor varieties (7963 and T8667) planted in the same orchard. Physiological characteristics were significantly affected by the delay in harvest periods, specifically the physicochemical properties of each coffee bean variety between the periods. For the green 7963 variety, the defect rate decreased from 11.08% to 4.19% while chlorogenic acid content increased from 3.78% to 4.99% as the harvest period was delayed. The 7963 variety harvested in February and March and T8667 variety harvested in February had the best quality performance, and their cupping scores were significantly higher than those harvested in other periods. Furthermore, a high correlation was found between physical attributes (defect rate, thousand-grain weight, and green bean size), chemical components (lipids, proteins, chlorogenic acid, caffeine, and trigonelline) and cupping scores.

## 1. Introduction

Coffee (*Coffea* sp.) is an important tropical crop, and its beverage is prepared from ground roasted beans. The sense of pleasure, satisfaction, and relaxation provided by coffee consumption—along with its demonstrated effects on mood enhancement, psychomotor activation, and cognitive performance—has promoted its evolution from a traditional beverage into an essential daily item in numerous cultures and communities [1,2]. Although China has a profound heritage of tea drinking, coffee is becoming increasingly popular nationwide. China’s coffee consumption has surged by nearly 150% over the past decade is projected to reach 6.3 million bags (60 kg) in 2025 [3]. This phenomenon could be linked to the accelerated rhythm of life in modern Chinese cities, leading to transformed perceptions and consumption patterns of coffee, especially among the youth demographic. At present, the predominant coffee-growing areas in China are concentrated in Yunnan Hainan and Guangxi Provinces located between 25° N and 30° S latitude, a region globally recognized as the “Coffee Golden Belt”.

Commercial coffee varieties primarily consist of Arabica coffee (*Coffea arabica* L.) and Robusta coffee (*Coffea canephora* L.). Catimor is a variety of Arabica originating from a cross between Caturra and Timor. Specifically, Catimor 7963 (7963) is a six-generation breeding product of Caturra 19/1 and HDT 832/1, whereas Catimor T8667 (T8667) was selected from a hybrid of Timor Hybrid 832/1 and Caturra [4]. 7963 and T8667, which currently constitutes >80% of the coffee planting area in Guangxi, are the dominant locally adapted cultivars representing regional characteristics. However, due to the relatively brief history of coffee cultivation in Guangxi, comparative studies with countries where coffee production is a primary agricultural activity face challenges such as limited germplasm diversity, less advanced management practices, and imprecise harvest timing.

Coffee beans are mainly characterized by their physical properties (mass, defects and particle size distribution), chemical components (chlorogenic acid, proteins, lipids, caffeine, trigonelline and other substances) and cup quality (sensory characteristics). These characteristics are significantly affected by multiple factors including genetics, altitude, climate and soil characteristics [5,6,7,8]. Chemical components in green coffee beans—such as caffeine, trigonelline, and chlorogenic acid—are recognized as flavor precursor compounds that impact the flavor, aroma, and bitterness of brewed coffee. Coffee bean quality, defect levels, and particle size distribution influence roasting uniformity and the taste of the final brew. The complex interactions between these physicochemical properties ultimately determine the quality of coffee beverages, a critical characteristic shaping consumer preferences and economic value. Most current studies have focused on the impacts of environmental conditions, brewing parameters (methods, water properties such as pH, hardness, and mineral composition), roasting, and processing on physicochemical properties and cup quality [9,10,11,12], while few have explored changes in these parameters across consecutive coffee fruiting seasons. Previous reports have indicated that coffee harvest period can have a major impact on its quality [13,14], likely due to the temperate and ecological variables that differ among the periods. Furthermore, studies suggest that crop quality characteristics may be differentially affected by harvest period among coffee bean varieties. This phenomenon, resulting from the interaction between genetic and environmental factors, is common in many agricultural plants such as avocados [15], figs [16], and olives [17]. For all these reasons, it is important to take coffee bean variety into consideration when investigating the impact of harvest periods on coffee quality.

This study examined in detail the physicochemical properties and sensory characteristics of two coffee bean varieties, 7963 and T8667, which are grown under the unique climatic and soil conditions of Xilin County, Baise City, Guangxi Zhuang Autonomous Region, China. The primary objective was to evaluate the quality characteristics of coffee beans harvested at different periods and to determine the optimal harvest time for obtaining fruits with the highest beneficial qualities. Our findings provide an empirical basis for determining the optimal harvest time period for growing and harvesting these bean varieties in order to harness their full potential.

## 2. Materials and Methods

### 2.1. Samples

Two different varieties of Catimor coffee (7963 and T8667) were planted in the same commercial plantation (altitude = 900 m) in Xilin County, Baise City, Guangxi Zhuang Autonomous Region, China, under uniform agronomic management practices. The soil at the plantation was identified as loam, with organic matter content ranging from 12.1 to 17.0 mg/kg and pH values between 5.11 and 5.21. Fully ripe coffee cherries were manually harvested in four different harvest periods—10 December 2024 (Harvest Period 1), 10 January 2025 (Harvest Period 2), 10 February 2025 (Harvest Period 3), and 10 March 2025 (Harvest Period 4). After harvest, cherries were subjected to a flotation process to remove unripe fruits, followed by wet processing involving immediate dehulling and fermentation in water at room temperature for 3 days. Fermented beans were then washed with clean water and sun-dried until they attained ~12% moisture content.

### 2.2. Standards and Chemicals

Chlorogenic acid (CGA), caffeine, and trigonelline standards were purchased from Desite Biotechnology Co., Ltd. (Chengdu, China). Other reagents used (phosphoric acid, acetonitrile) were of analytical grade and obtained from Thermo-Fisher Scientific, Inc. (Shanghai, China). Ultrapure water (18.2 MΩ·cm, pH 6.8–7.2) was prepared with a Master-S15 (Hitech Instrument Co., Ltd., Shanghai, China) to minimize variability and ensure consistency in experimental results.

### 2.3. Climatic Data

Climatic variables including precipitation, average temperature and average minimum and maximum temperature range were obtained from the National Meteorological Science Data Center of China. Climatic data for one month prior to each harvest period were collected in this study (Table 1).

### 2.4. Characterization of Green Coffee Bean Physicochemical Properties

Green coffee beans were analyzed to determine defect rate, thousand-grain weight, CGA content, caffeine content, trigonelline content, protein content, lipid content, pH value and total dissolved solids (TDS). All experiments were performed in triplicate.

Defect rate was measured according to the method of Kath et al. [18]. The percentage of total defective beans was calculated from 300 g of beans to calculate defect rate as a fraction of mass.

Thousand-grain weight of the beans was measured using an FA2204E electronic analytical balance (XingYun Electronic Equipment Co., Ltd., Changzhou, China).

Bean particle sizes were assessed as described by Brighenti et al. [19]. In brief, ~300 g of coffee beans were passed through a series of sieves with descending mesh sizes—20 mesh (7.5 mm), 18 mesh (7.1 mm), 16 mesh (6.3 mm), and 14 mesh (5.6 mm). The retention percentage on each sieve was evaluated separately to classify the particle sizes.

Coffee bean CGA, caffeine, and trigonelline content were analyzed according to the method of Getachew et al. [8], with slight modifications. Briefly, 0.5 g of ground beans was placed in 80 mL of 0.1% phosphoric acid aqueous solution (*v*/*v*) and extracted in a boiling water bath for 30 min with stirring. After cooling, the extract was adjusted to a final volume of 100 mL with 0.1% phosphoric acid aqueous solution (*v*/*v*). The diluted extract (1 mL) was filtered through a 0.22 μm polytetrafluoroethylene (PTFE) membrane prior to injection into a Waters e2695 High Performance Liquid Chromatography (HPLC) column (Waters Co., Shanghai, China). Chromatographic separation was performed on a C18 column (250 × 4.6 mm, 5 μm i.d.) maintained at 30 °C. The mobile phase was composed of 0.1% phosphoric acid aqueous solution (*v*/*v*) and acetonitrile in a ratio of 80:20 (*v*/*v*). The isocratic elution was carried out at a flow rate of 1.0 mL min^−1^ with an injection volume of 10 μL. Caffeine and trigonelline were detected at 254 nm, while CGA was detected at 320 nm using an ultraviolet (UV) detector. The reported CGA content represented the total chlorogenic acid content. Standard curves were constructed using CGA, caffeine, and trigonelline standards. Results were expressed as the % dry basis (%, d.b.) concentration.

Coffee bean protein and lipid contents were determined according to the methods of AOAC [20]. The results were expressed as %, d.b. concentration.

Coffee bean samples were analyzed for acidity (i.e., pH) and total dissolved solids (TDS) content as in previous studies [21,22]. Briefly, a sample of 2.75 g of ground beans which had passed through a 30-mesh sieve was immersed in 50 mL of hot ultrapure water with stirring. After the mixture was cooled to room temperature, pH was measured using a pH meter (Leici PHS-3C, Shanghai, China), and TDS content was determined using a coffee concentration meter (VST LAB Coffee III, Waters Co., Santa Barbara, CA, USA). Results were expressed as the ratio (%) of the mass of dissolved substances to the total mass of the beverage.

### 2.5. Bean Roasting, Grinding, and Brewing

A sample of 300 g beans from each sample was put into the roasting machine (ACR002A-050, Mita Life Electrical Appliances Co., Ltd., Foshan, Guangdong, China) and carefully roasted at 200 °C for 7–8 min to achieve a medium roast (Agtron value 55–60) [8]. After roasting, the beans were stored in coffee-specific one-way de-gassing bags for 72 h.

Immediately prior to brewing, roasted beans were ground to the same consistency using a coffee grinder (TEGB01S, Timemore Coffee Ware Co., Ltd., Shanghai, China). Exactly 8.25 g of roasted coffee powder was then poured into 150 mL of hot water at 93 °C, stirred until uniform, and then left to stand. After 3 min, the mixture was filtered through filter paper, cooled to room temperature, and the resulting filtrate was the coffee brew.

### 2.6. Characterization of Roasted Coffee Bean Physicochemical Properties and Cupping Quality

Using coffee brew, roasted coffee beans were analyzed to determine CGA content, caffeine content, trigonelline content, pH value and TDS according to nearly identical methods outlined in Section 2.2 above. All experiments were made in triplicate.

To assess cupping quality, exactly 8.25 g of roasted coffee powder was poured into 150 mL of hot water at 93 °C, stirred until uniform, and then left to stand. After 3 min, the floating grounds were skimmed off and cupping evaluation was conducted by a panel of experts comprised of 10 internationally trained and certified Q-graders, each with at least one year of experience in coffee sensory evaluation, that assessed the quality according to the cupping system of the Specialty Coffee Association (SCA). The evaluation was conducted in triplicate with samples presented at room temperature in a randomized order. Each cupper made independent judgments using a cupping survey form. Results were expressed as the average score of the 10 cuppers [8].

### 2.7. Statistical Analysis

One-way analysis of variance (ANOVA), two-way ANOVA, correlation analysis and hierarchical cluster analysis (HCA) were performed using IBM SPSS Statistics 26 (29 Inc., Chicago, IL, USA). For one-way ANOVA, Duncan’s test was applied to compare means at the 5% significance level, with statistical significance defined as *p* < 0.05. Two-way ANOVA was employed to evaluate the influence of varieties, harvest time, and their interactions on the physicochemical properties and sensory characteristics of the coffee beans. HCA was performed using the between-groups linkage method, with Euclidean distances as the dissimilarity coefficient.

## 3. Results and Discussion

### 3.1. Green Coffee Bean Characteristics

#### 3.1.1. Analysis of Defect Rate, Thousand-Grain Weight, pH, and TDS

Table 2 shows the characterization of defect rate, 1000-grain weight, pH, and TDS of green coffee bean samples from different varieties and harvest periods. Defects mainly included hollow, insect-damaged, broken, and discolored beans. As shown in Table 2, the defect rate ranged from 2.1% to 7.48%, which fell within the ranges reported by Worku et al. [23]. Lower defect rates were observed in both varieties at harvest periods 3 and 4 (5.6% and 4.19% for 7963 variety; 4.72% and 6.78% for T8667 variety). Certain specific chemical components in defective coffee beans can induce off-flavors after roasting, thereby reducing the sensory characteristics and quality of coffee beverages. Chemicals such as 1-methylpyrrole, 5-methyl-2-furfurylfuran, and 2-methylfuran are exclusively present in defective roasted coffee beans [24]. Furthermore, green immature defective seeds are rich in 2-methylpyrazine and 2-furfuryl acetate, while butyrolactone and other chemicals are mainly present in green acidic seeds [25]. The highest defect rates for both 7963 and T8667 varieties occurred at 7% and 7.48%, respectively, in harvest period 2, potentially due to the extreme weather conditions in January. The average temperature during harvest period 2 (6.8–14 °C; Table 1) was substantially below the optimal range for coffee growth (18–23 °C) [26]. Prolonged exposure to suboptimal chilling temperatures might inhibit the growth of coffee cherries and reduce bean quality as reported by Tolessa and colleagues [14]. Furthermore, at temperatures below 15 °C, Arabica coffee becomes more susceptible to wilt disease caused by Fusarium xylarioides as well as coffee berry disease caused by Colletotrichum kahawae, which may contribute to a higher incidence of defective beans [27,28]. This is likely owing to the fact that low-temperature environments not only directly favor the development of pathogens but may also slow the physiological metabolism of coffee berries, thereby reducing their innate resistance and indirectly elevating the risk of infection by fungi such as Fusarium xylarioides and Colletotrichum kahawae. The T8667 variety exhibited a higher defect rate than the 7963 variety across most harvest periods—though the difference was not statistically significant—and its defect rate also showed greater variability in response to temperature changes. These findings suggest that T8667 is more susceptible to low-temperature stress and consequently more vulnerable to infection by coffee pathogenic fungi. Another important environmental variable impacted by seasons is water supply. Given the substantial increase in water demand during the ripening and harvesting stages of coffee fruits [7], water supply is a critical factor that can influence coffee quality during these stages. Studies have also shown that high fruit set, peak photosynthetic rates, and sustained fruit size are all heavily influenced by water supply [29]. Coffee phenology is clearly sensitive to both the amount and timing of precipitation. Compared to other harvest periods, the precipitation during harvest period 2 was 0 mm (Table 1), inducing water stress in the plants during the ripening and harvesting stages. This stress inhibited fruit growth and filling, ultimately increasing the incidence of hollow beans.

The thousand-grain weight of 7963 variety ranged from 120.89 to 139.07 g, compared with 111.88 to 157.63 g for T8667 (Table 2). These values are slightly lower than the thousand-grain weight ranges reported by Tolessa et al. [14]. Notably, both varieties exhibited the same trend in thousand-grain weight and defect rate, with the minimum thousand-grain weight also recorded in harvest period 2 (120.89 g for 7963 variety; 111.88 g for T8667 variety). We propose that this was also influenced by low temperatures and water stress. Williams et al. [30] and Kath et al. [18] demonstrated that temperature and rainfall significantly affect coffee bean yield and quality of coffee beans. Specifically, both low temperature and water stress compromise coffee growth and maturation, increase the probability of branch death and leaf abscission, and ultimately reduce bean size and integrity. After harvest period 2, the thousand-grain weight increased with later harvests. T8667 variety showed greater range in thousand-grain weight (111.88 g to 157.63 g) compared with 7963 (120.89 g to 139.07 g). Two-way ANOVA (Table 3) also confirmed a highly significant interaction (*p* < 0.01) between harvest period and variety on the thousand-grain weight. This indicates that T8667 variety is more susceptible to low temperature and water stress compared with 7963.

The pH of 7963 variety ranged from 6.09 to 6.16 (Table 2), and the maximum pH (6.16) was observed at harvest period 3. No significant differences were observed among the other harvest periods. The pH of T8667 variety ranged from 5.96 to 6.22, with the minimum (5.96) in harvest period 3 and maximum (6.22) in period 4. No significant differences in pH between harvest period 1 and harvest period 2 were observed. Both malic and citric acid contents contribute to the acidity of coffee beverages [31]. Our results suggest that the effects of different harvest periods on coffee bean organic acid contents may vary, and further research is needed to clarify the specific mechanisms. Furthermore, previous studies have shown that air temperature and rainfall affect the acidity of green coffee beans [32]. However, the present study found that the impact of climate on coffee bean pH is variable.

TDS of the green coffee beans in the present study ranged from 1.73% to 2.37% for 7963, and 1.67% to 3.5% for T8667 (Table 2). For specialty coffee, a higher TDS content is required for robust industrial yield and beverage quality. A such, TDS is frequently viewed as proportional to coffee cup quality [33]. Two-way ANOVA (Table 3) confirmed that bean pH and TDS were significantly (*p* < 0.01) influenced by harvest period and the interaction between variety and harvest period. The variety exerted significant influence only on TDS.

#### 3.1.2. Analysis of Particle Size Distribution

**Figure 1:**
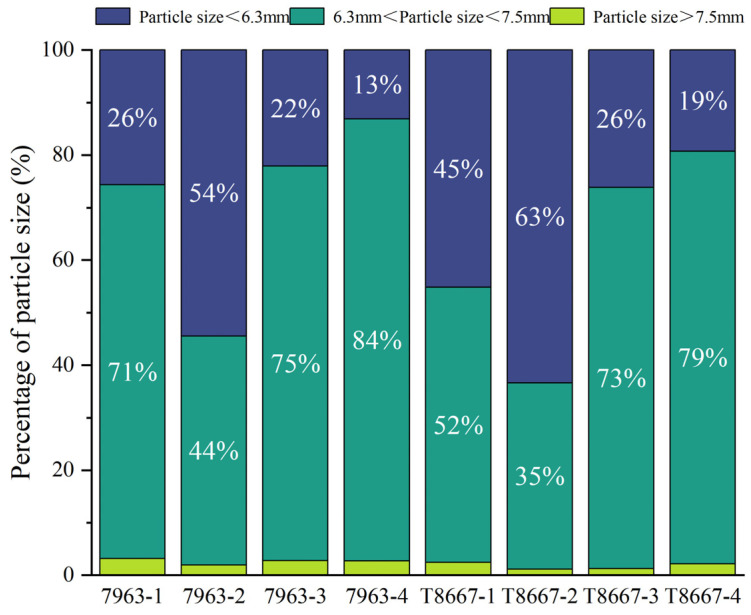
Particle size proportion of green coffee beans at different harvest periods.

Particle size distributions are presented in Figure 1. Notably, only in harvest period 2 did the proportion of green coffee beans with a particle size of <6.3 mm exceed that of those with a particle size of 6.3–7.5 mm. This indicates that there was a high proportion of small beans and a non-uniform size distribution in harvest period 2. Bean particle size can influence market value, with larger beans commanding premium prices within a given variety. However, bean size uniformity is another parameter that impacts coffee quality and flavor [34], as homogeneous bean sizes ensure roast consistency. Over-roasted small beans impart smoky notes and under-roasted large beans impart grassy flavors, both compromising beverage quality. Notably, in this study, the particle size distribution of T8667 displayed suboptimal uniformity during harvest period 1, with the proportion of beans with a particle size of <6.3 mm and those with 6.3–7.5 mm range being roughly equivalent (45% and 52%, respectively). This may imply suboptimal value and roasting performance for this variety in harvest period 1. Bean uniformity progressively improved with later harvest periods, reaching 79% of beans in the 6.3–7.5 mm range for T8667 during period 4. 7963 variety also exhibited a similar trend with less pronounced variation, consistent with the results observed for the thousand-grain weight of the two varieties. Furthermore, within each harvest period, 7963 consistently maintained higher proportions of beans in the 6.3–7.5 mm range compared with T8667.

#### 3.1.3. Analysis of Lipid and Protein Contents

Lipids are essential chemical determinants of quality and flavor in green coffee beans. During roasting, they participate in decomposition and auto-oxidation reactions, releasing volatile compounds (e.g., aldehydes, ketones, and alcohols) that contribute to coffee flavor and aroma [35]. In this study, the % dry basis lipid contents ranged from 13.34% to 14.16% for 7963 variety, and 13.29% to 13.62% for T8667 (Table 4), in agreement with reported values (10–15%) for green Arabica coffee beans [36,37]. Lipid contents of both varieties progressively increased with later harvests. A delayed harvest period is a consequence of slower coffee bean maturation, allowing for better bean filling and more lipid accumulation [14]. Two-way ANOVA (Table 3) confirmed significant (*p* < 0.01) main effects of harvest period, variety, and their interaction on lipid content of the beans.

Proteins contribute to coffee flavor and aroma through Maillard reactions during roasting. Here, protein content in 7963 ranged from 12.53% to 14.26%, and 11.21% to 13.43% for T8667 (Table 4). These values are also in agreement with reported Arabica bean protein content (11–15.66%) [35,38]. The average protein content of 7963 was significantly higher than that of T8667 (*p* < 0.05), suggesting that genetic factors can affect the protein content. Two-way ANOVA (Table 3) also revealed that protein content was significantly (*p* < 0.01) influenced by harvest period, variety, and their interaction. Similar to lipids, the protein content of both varieties seemed to increase with later harvest periods. A fluctuation was observed in 7963 at harvest period 4 with a slight decrease (from 14.26% to 13.96%), possibly owing to the increase in temperature during harvest period 4. Several reports have indicated that coffee bean protein content is susceptible to ambient temperature, with even small temperature changes exerting a significant impact [33,39]. According to meteorological data, the average temperature during harvest period 4 was 20.8 °C (Table 1), which was 5.4 °C higher than harvest period 3 (15.4 °C). This may have contributed to the slight decrease in 7963 protein content during the final harvest period.

#### 3.1.4. Analysis of Chlorogenic Acid, Caffeine and Trigonelline Content

Chlorogenic acid (CGA), caffeine, trigonelline, and related chemicals in green coffee beans serve as key components in brewed coffee flavor and aroma, particularly bitter flavor [8]. In this study, the CGA content in 7963 ranged from 3.76% to 4.99%, while that of T8667 ranged from 3.39% to 3.74% (Table 4). CGA accumulation progressively increased in 7963 with later harvest periods, whereas T8667 showed no significant difference across the periods. This demonstrates that harvest period differentially affects CGA content in green coffee beans based on underlying genetics (i.e., variety). There was also a significant difference in average CGA content between the two varieties, with 7963 showing a significantly higher CGA content than T8667 (4.2% vs. 3.53%; *p* < 0.05). This could be attributed to the application of different post-harvest processing techniques, environmental factors, and genetic variations among varieties, as has been previously reported [13]. Two-way ANOVA (Table 3) also confirmed significant (*p* < 0.01) effects of harvest period, variety, and their interaction on CGA content. The observed increase in CGA content during later harvests could be attributed to the slowed maturation process of the beans, allowing more time for bean filling, a finding consistent with Arévalo et al. [13] Additionally, weather factors may also affect green coffee bean CGA content. Previous studies have demonstrated that high water deficit and temperature can increase green coffee bean acidity [33].

Caffeine and trigonelline are two important alkaloid compounds in coffee. As the most abundant alkaloid, caffeine is synthesized in immature fruits and accumulates during coffee bean development. It plays a protective role in coffee growth, protecting coffee seedlings from pests and pathogens. Caffeine can also leach into the soil to reduce the germination of neighboring seeds, thereby endowing a competitive advantage to the coffee by creating a more favorable microbiological and physicochemical environment for growth [31,40]. Caffeine and CGA have direct impact on human health and are key biochemical determinants of coffee beverage quality [14]. In this study, caffeine content in 7963 ranged from 0.87% to 1.38%, while that of T8667 ranged from 0.89% to 1.15% (Table 4), consistent with the range reported by Mehari et al. [41] in a similar study (0.87–1.38%). There were no significant differences in caffeine content between the two varieties (*p* > 0.05). It is noteworthy that the caffeine content of 7963 was consistently higher than that of T8667 at each harvest period, although the difference was not statistically significant. This may suggest that 7963 possesses superior insect resistance compared to T8667, a finding consistent with the defect rate observed in green coffee beans (Table 2). Furthermore, both varieties exhibited progressive caffeine accumulation with later harvests. This also may be attributed to the longer maturation period, which provides additional time for caffeine accumulation during development [34].

Trigonelline is the second most abundant alkaloid in coffee. It is an alkaloid produced by the enzymatic methylation of nicotinic acid, and its content in green coffee beans is only slightly less than caffeine [41]. In this study, trigonelline content in was differentially impacted by harvest period between the two varieties. Trigonelline content in 7963 increased during harvest periods 3 and 4, whereas T8667 showed no significant difference across the four harvest periods. However, there was no significant difference in mean trigonelline content between varieties. Barbosa et al. [31] found that caffeine and trigonelline contents are highly influenced by genotype and environmental conditions. This is not entirely consistent with the present findings. Two-way ANOVA (Table 3) showed that variety had no significant effect on the caffeine content in green coffee beans (*p* > 0.05). Furthermore, the interaction between variety and harvest period also had no significant effect on the contents of major alkaloids (caffeine and trigonelline) in the beans (*p* > 0.05). Only the contents of major alkaloids in the beans was impacted by harvest period (*p* < 0.05).

### 3.2. Characteristics of Roasted Coffee Beans

#### 3.2.1. Sensory Analysis

**Figure 2:**
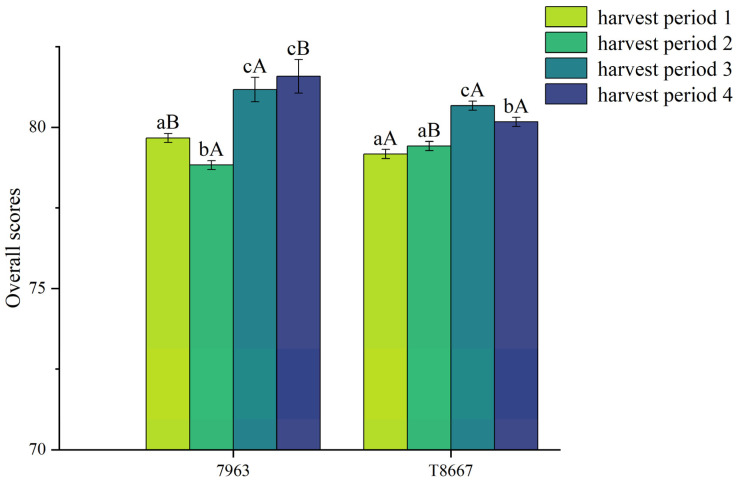
Total cupping scores of coffee beans at different harvest periods. Different lowercase letters indicate significant differences among samples of the same vareity analyzed at different harvest periods (*p* < 0.05). Different uppercase letters indicate significant differences between varieties at the same harvest period (*p* < 0.05).

Total coffee cupping scores of all samples are presented in Figure 2. Two-way ANOVA confirmed significant effects (*p* < 0.05) of variety, harvest period, and their interaction on cupping scores. For 7963, samples from harvest periods 3 and 4 obtained the highest cupping scores (81.17 and 81.58, respectively), while those from harvest period 2 received the lowest scores (78.83). As noted by the cuppers, samples of 7963 from harvest periods 3 and 4 exhibited cocoa, nutty, and chocolate flavors, while samples of this variety from harvest period 2 presented unpleasant flavors such as cut tobacco. For T8667, samples from harvest period 3 obtained the highest scores (80.67), whereas those from harvest periods 1 and 2 received the lowest scores (79.17 and 79.42, respectively). T8667 samples from harvest period 3 were described as having berry, tropical fruit, and wine-filled chocolate flavors, while samples from harvest periods 1 and 2 presented unpleasant flavors such as green beans and barley tea. Based on the cupping evaluation results, it seems the optimal harvest period for the 7963 is February and March, while February seems optimal for T8667 given that there was a significant cupping score reduction in March for this variety (80.67 to 80.17). However, there were no significant differences in average cupping scores between the two varieties. The observed flavor variations may be associated with biochemical changes within the beans across harvesting periods, which require further research to fully delineate.

#### 3.2.2. Analysis of pH and TDS

The pH of roasted beans in our study ranged from 5.14 to 5.32 (Table 5), which was significantly lower than green coffee beans. These results are in line with those of previous studies which have demonstrated that roasted coffee beans have lower pH owing to the degraded acids formed during the roasting process [42].

TDS of roasted coffee bean samples ranged from 1.27% to 1.58% (Table 5), aligning with Specialty Coffee Association of America (SCAA) recommendations (0.79–1.38%) [43]. Furthermore, the variation of these parameters in roasted coffee beans were similar to those of green coffee beans, and a significant positive correlation was observed between pH and TDS of roasted beans and green coffee beans (Table 5). Two-way ANOVA (Table 3) revealed significant effects (*p* < 0.01) of variety, harvest period, and their interaction on roasted bean pH. In contrast, TDS in the roasted beans was not significantly affected by these factors (*p* > 0.05).

#### 3.2.3. Analysis of CGA, Caffeine and Trigonelline Content

CGA represents a group of multifunctional phenolic compounds that are degraded into caffeic acid, lactones, and other phenolic derivatives through Maillard and Strecker reactions during roasting, thereby increasing bitterness, astringency, and aroma [34]. Pereira et al. [44] reported that C18:4-vinyl-3-methoxyphenol or 4-vinylguaiacol is a degradation product of CGA, among which 4-ethylguaiacol has a pungent phenolic aroma. The degradation rate of CGA depends on roasting intensity, with a loss of approximately 60% during light roasting and almost 100% during deep roasting [45]. In our medium-roasted samples, CGA ranged from 1.09% to 1.67% (Table 5). In general, the roasted bean CGA content mirrored a similar trend found in their respective green beans. This may be attributed to the fact that the roasting method used in this study was medium roasting, where the CGA loss rate was equivalent between the varieties.

Caffeine exhibits thermal stability during roasting, maintaining relatively constant concentrations without affecting aroma development. Roasting-induced structural and biochemical changes of coffee beans—including moisture loss, volume expansion, and pore formation—are known to enhance caffeine extractability [40]. In contrast, heat-labile trigonelline degrades during roasting, leading to the formation of sensory-related volatile compounds (e.g., pyrroles and pyridines) while undergoing demethylation to yield physiologically active nicotinic acid, a small molecule which modulates energy metabolism and neural function in humans [5]. In this study, the caffeine and trigonelline contents in roasted coffee bean samples ranged from 0.75% to 0.85% and 0.35% to 0.42%, respectively (Table 5). Moreover, variation in this content in roasted beans was mostly similar to those in green beans.

**Figure 3:**
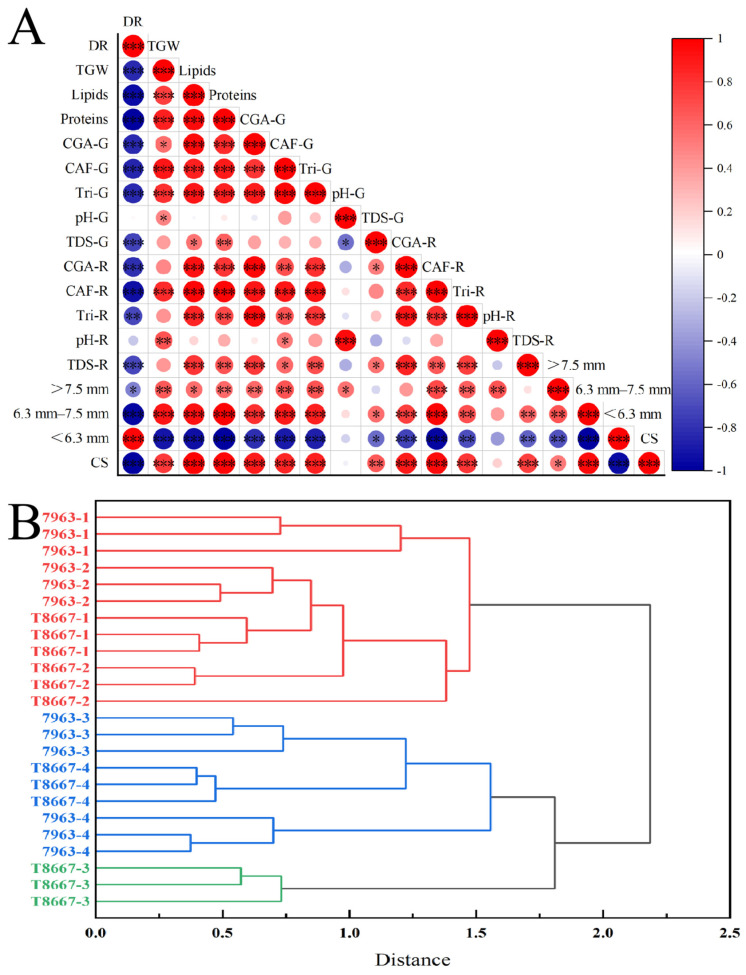
Pearson correlation analysis plot (**A**) and dendrogram of the hierarchical cluster analysis (**B**) of coffee bean parameters: Defect rate (DR), Thousand-grain weight (TGW), Chlorogenic acid content of green beans (CGA-G), Caffeine content of green beans (CAF-G), Trigonelline of green beans (Tri-G), pH of green beans (pH-G), TDS of green beans (TDS-G), Chlorogenic acid content of roasted beans (CGA-R), Caffeine content of roasted beans (CAF-R), Trigonelline of roasted beans (Tri-R), pH of roasted beans (pH-R), TDS of roasted beans (TDS-R), Cupping scores (CS). *p*-value: * *p* < 0.05, ** *p* < 0.01, *** *p* < 0.001.

A significant positive correlation (Figure 3A) between roasted and green beans was found for CGA, caffeine, and trigonelline content. Two-way ANOVA (Table 3) confirmed significant effects (*p* < 0.05) of variety, harvest period, and their interaction on these compounds in roasted beans.

#### 3.2.4. Correlation and Hierarchical Cluster Analysis

Coffee beverage quality is determined by flavors developed during roasting, where compounds in unroasted coffee beans are influenced chiefly by chemical and physical reactions during bean development and maturation. In this study, a significant (*p* < 0.05) positive correlation was observed (Figure 3A) between cupping scores and multiple physicochemical parameters including thousand-grain weight, lipid, protein, CGA, caffeine, and trigonelline contents in both green and roasted beans; TDS in both green and roasted beans; and particle sizes > 7.5 mm and between 6.3–7.5 mm in green beans. Conversely, the cupping scores were negatively correlated (*p* < 0.05) with both the defect rate and particle size < 6.3 mm. Green and roasted coffee beans from later harvest periods (i.e., 3 and 4) generally exhibited higher lipid, protein, CGA, caffeine, and trigonelline content, and achieved higher cupping scores. Furthermore, hierarchical cluster analysis (HCA) assembled the samples according to their physicochemical properties and sensory characteristics (Figure 3B). The clustering pattern illustrates the substantial influence of harvest period on coffee quality parameters. In HCA, both varieties across the harvesting periods were clustered into 3 categories. Among these, early-harvest coffees (December and January) from both varieties were classified into one category, suggesting that the early-harvested coffees have similar physicochemical and sensory properties that render less desirable quality and lower cupping scores. Notably, late-harvest coffees (February and March) were grouped into one category, with exception of T8667 harvested in period 3 (February). Both clusters achieved relatively high cupping scores despite limited physicochemical similarity, suggesting the highest-scoring samples across varieties possess distinct physicochemical profiles. These results demonstrate that complex interactions among compounds in coffee differentially modulate sensory outcomes, potentially explaining T8667-3′s anomalous positioning despite high cupping scores.

As indicated in Figure 3A, the defect rate and proportion of particle size < 6.3 mm were negatively correlated with all other physicochemical properties, except for the green and roasted bean pH. This suggests that higher defect rates and a larger proportion of particle size < 6.3 mm in green beans may reduce the content of key flavor precursors such as lipids, proteins, CGA, caffeine, and trigonelline, ultimately leading to a lower coffee cupping scores. Conversely, thousand-grain weight was positively correlated with the contents of numerous flavor precursors. This finding aligns with those of Bote et al. [46], who reported a positive correlation between coffee bean quality characteristics and thousand-grain weight. This result might be explained by the fact that factors which promote coffee bean quality also facilitate the accumulation of dry matter in the beans, thereby increasing thousand-grain weight. Similarly, Marie et al. [47] found a positive correlation between 100-grain weight of coffee beans and total SCA sensory scores. Collectively, these findings suggest that physical properties of green coffee beans (e.g., defect rate, particle size distribution, 1000-grain weight) are strongly associated with their chemical composition and sensory quality attributes. These physical metrics represent external manifestations of intrinsic coffee quality and can serve as biomarkers by which growers can preliminarily assess internal chemical content and quality potential.

In terms of chemical components, the diverse array of lipids (52 types) in roasted beans contributes positively to key sensory attributes of coffee beverages, including flavor, color, and foam stability [48]. Our results are similar to Franca et al. [49], who found that higher lipid content in green coffee beans produced better coffee cupping quality.

During roasting, proteins and reducing sugars undergo Maillard reactions, generating color and aroma precursors such as pyridine, pyrrole, pyrazine, thiazole, and ketone [48] which have been shown to contribute to color and aroma in coffee beverages. However, the reported impact of protein content on overall coffee sensory evaluation remains equivocal. Barbosa et al. [31] associated high protein content with lower cupping quality, while other studies have reported a positive correlation between protein content and coffee cupping scores [35,49], in agreement with our findings (Figure 3A). This inconsistency may be due to variations in specific protein content and their amino acid composition within the beans, and their complex interactions with gustatory receptors may differentially influence the perceived flavor of coffee beverages.

CGA undergoes numerous chemical reactions during roasting, including lactonization, epimerization, and isomerization. These reactions impart bitterness, astringency, and acidity to brewed coffee. Some studies suggest that various chlorogenic acid subclasses become oxidized during storage and degrade during roasting, forming off-flavor compounds that impart unpleasant sensory attributes and reduce coffee beverage quality [40,50]. In contrast to these reports, our study found a positive correlation between CGA content (in both green and roasted beans) and cupping scores (Figure 3A). This apparent discrepancy can be explained as follows: (1) Bitterness and astringency are important attributes of coffee beverages [40,51], and the bitter and astringent substances produced by CGA during roasting are essential components required for high-quality brewed coffee; (2) Many low-molecular-weight compounds formed by the decomposition of CGA during roasting participate in melanoidin formation, which contributes to the development of coffee flavor and color [51]; (3) Studies have reported that certain chlorogenic acid derivatives function as coffee flavor modulators, significantly enhancing scores for flavor, aroma, aftertaste, acidity, body, balance, and overall impression [52]. (4) Most importantly, roasting conditions in this study were non-extreme, minimizing lactonization and decomposition pathways that generate off-flavors. Thus, it is likely that only a small portion of CGA underwent these detrimental reactions.

Caffeine stimulates the human central nervous system and contributes to the perceived bitterness and astringency of brewed coffee. In this study, samples from later harvest periods generally exhibited higher caffeine content yet received higher cupping scores—a finding that contradicts Barbosa et al. [31]. This discrepancy may arise from differential interactions between caffeine and other metabolites, which potentially modulate its impact on flavor perception [40]. Later harvest periods indicate longer coffee fruit ripening. The extended maturation likely allows accumulation of flavor-modulating compounds that mitigate caffeine’s bitterness, resulting in reduced perceived astringency. An additional complicating factor is that caffeine as a metabolite cannot be considered in isolation, as it may be linked to other metabolic pathways that correlate with coffee flavor attributes. More detailed studies are required to determine whether one or more of these compounds exert a direct causal influence on coffee flavor.

Trigonelline significantly contributes to the bitterness of brewed coffee [51] and strongly influences beverage quality. Both green and roasted coffee trigonelline content exhibited a strong positive correlation with cupping scores in this study, consistent with previous reports [49,50,53].

In summary, although caffeine and CGA contribute to coffee bitterness and astringency, their elevated concentrations in late-harvest samples did not impair beverage quality in this study. This suggests extended fruit development during later harvest periods enriches the composition and content of key metabolites (e.g., lipids, proteins) in coffee beans, and their synergistic interactions positively modulate sensory attributes triggered by the brewed beverages.

## 4. Conclusions

This study examined the response of two distinct coffee varieties to environmental factors across harvest periods. The findings highlight critical aspects that underlie the cultivation of premium-quality coffee fruits—information that will be vitally important for growers aiming to enhance coffee quality. Our analysis demonstrates that cupping scores are significantly affected by the interaction between coffee bean variety and harvest period factors. Higher-scoring samples were positively correlated with key chemical components—lipids, proteins, chlorogenic acid, caffeine, and trigonelline—and with physical properties including thousand-grain weight and particle sizes > 7.5 mm and 6.3–7.5 mm. Conversely, negative correlations emerged with defect rates and particles < 6.3 mm. Optimal harvest period varied by coffee bean variety. Specifically, periods 3–4 (February and March, respectively) were optimal for 7963 while period 3 (February) was optimal for T8667. Compared to period 3, T8667 harvested in period 4 exhibited reduced cupping scores, lower CGA and trigonelline concentrations, and elevated defect rates. Taken together, these results provide information for coffee producers to better manage the harvesting process and develop a wider range of coffee products, thereby improving quality of their products and enhancing market competitiveness.

## Figures and Tables

**Table 1:** Climatic data for each harvest period.

Harvest Periods	Average Temperature (°C)	Precipitation (mm)	Average Temperature Range (°C)
Harvest Period 1	19.4	15	11.7–19.8
Harvest Period 2	14.1	0	6.8–14
Harvest Period 3	15.4	30	9.4–21.1
Harvest Period 4	20.8	2.9	12.7–26.9

**Table 2:** Defect rate, thousand-grain weight, pH, and TDS of green coffee beans ^1^.

	7963	T8667
***Defective rate* (%) **		
Harvest period 1	11.08 ± 1.99 ^bA^	12.7 ± 1.59 ^bA^
Harvest period 2	14 ± 0.74 ^cA^	14.95 ± 0.48 ^cA^
Harvest period 3	5.6 ± 0.35 ^aA^	4.72 ± 0.75 ^aA^
Harvest period 4	4.19 ± 0.59 ^aA^	6.78 ± 0.85 ^bB^
mean	8.72 ± 4.28 ^A^	9.79 ± 4.45 ^A^
***Thousand-grain weight* (g) **		
Harvest period 1	128.84 ± 2.47 ^bB^	119.75 ± 3.44 ^bA^
Harvest period 2	120.89 ± 1.16 ^aB^	111.88 ± 0.99 ^aA^
Harvest period 3	133.15 ± 1.1 ^cB^	128.14 ± 1.38 ^cA^
Harvest period 4	139.07 ± 2.08 ^dA^	157.63 ± 0.63 ^dB^
mean	130.49 ± 7.09 ^A^	129.35 ± 18.15 ^A^
***pH***		
Harvest period 1	6.09 ± 0.03 ^aA^	6.13 ± 0.04 ^bA^
Harvest period 2	6.1 ± 0.01 ^aA^	6.09 ± 0.03 ^bA^
Harvest period 3	6.16 ± 0.01 ^bB^	5.96 ± 0.03 ^aA^
Harvest period 4	6.11 ± 0.02 ^aA^	6.22 ± 0.01 ^cB^
mean	6.11 ± 0.03 ^A^	6.1 ± 0.1 ^A^
***TDS* (%) **		
Harvest period 1	1.73 ± 0.12 ^aA^	1.7 ± 0.1 ^aA^
Harvest period 2	1.77 ± 0.06 ^aA^	1.67 ± 0.06 ^aA^
Harvest period 3	2.37 ± 0.06 ^bA^	3.5 ± 0.1 ^cB^
Harvest period 4	1.79 ± 0.06 ^aA^	2.1 ± 0.1 ^bB^
mean	1.91 ± 0.28 ^A^	2.24 ± 0.78 ^A^

^1^ Different lowercase letters indicate significant differences between harvest periods among samples of the same variety (*p* < 0.05). Different uppercase letters indicate significant differences between the two varieties at the same harvest period (*p* < 0.05).

**Table 3:** Significance values of two-way ANOVA for all analyzed variables ^1^.

Variables		Variety	Harvest Period	Interaction
Green beans	Defect rate	*	**	ns
	Thousand-grain weight	ns	**	**
	Chlorogenic acid	**	**	**
	Caffeine	ns	*	ns
	Trigonelline	ns	*	ns
	Lipids	**	**	**
	Proteins	**	**	**
	pH	ns	**	**
	TDS	**	**	**
Roasted beans	Chlorogenic acid	**	*	**
	Caffeine	**	**	**
	Trigonelline	**	**	**
	pH	**	**	**
	TDS	ns	ns	ns
	Cupping scores	**	**	**

^1^ *p*-value: ns: no significant; * *p*  <  0.05; ** *p*  <  0.01.

**Table 4:** Lipid, protein, CGA, caffeine and trigonelline contents in green coffee beans ^1^.

	7963	T8667
***Lipid (% d.b.)***		
Harvest period 1	13.46 ± 0.08 ^aB^	13.29 ± 0.07 ^aA^
Harvest period 2	13.34 ± 0.08 ^aA^	13.44 ± 0.07 ^bA^
Harvest period 3	13.78 ± 0.09 ^bA^	13.62 ± 0.05 ^cA^
Harvest period 4	14.16 ± 0.07 ^cB^	13.6 ± 0.05 ^cA^
mean	13.69 ± 0.34 ^A^	13.49 ± 0.15 ^A^
***Protein (% d.b.)***		
Harvest period 1	12.53 ± 0.05 ^aB^	11.36 ± 0.05 ^aA^
Harvest period 2	12.60 ± 0.07 ^aB^	11.21 ± 0.1 ^aA^
Harvest period 3	14.26 ± 0.06 ^cB^	13.33 ± 0.05 ^bA^
Harvest period 4	13.96 ± 0.08 ^bB^	13.43 ± 0.2 ^bA^
mean	13.34 ± 0.82 ^B^	12.33 ± 1.1 ^A^
***CGA (% d.b.)***		
Harvest period 1	3.78 ± 0.27 ^aA^	3.39 ± 0.26 ^aA^
Harvest period 2	3.76 ± 0.37 ^aA^	3.51 ± 0.25 ^aA^
Harvest period 3	4.28 ± 0.26 ^bA^	3.74 ± 0.31 ^aA^
Harvest period 4	4.99± 0.03 ^cB^	3.48 ± 0.32 ^aA^
mean	4.2 ± 0.57 ^B^	3.53 ± 0.28 ^A^
***Caffeine (% d.b.)***		
Harvest period 1	0.89 ± 0.09 ^aA^	0.86 ± 0.07 ^aA^
Harvest period 2	0.94 ± 0.1 ^aA^	0.84 ± 0.1 ^aA^
Harvest period 3	1.04 ± 0.12 ^abA^	0.92 ± 0.13 ^aA^
Harvest period 4	1.15 ± 0.08 ^bA^	1.14 ± 0.06 ^bA^
mean	1.01 ± 0.13 ^A^	0.94 ± 0.15 ^A^
***Trigonelline (% d.b.)***		
Harvest period 1	0.8 ± 0.08 ^aA^	0.81 ± 0.08 ^aA^
Harvest period 2	0.82 ± 0.09 ^aA^	0.78 ± 0.09 ^aA^
Harvest period 3	0.91 ± 0.11 ^abA^	0.82 ± 0.12 ^aA^
Harvest period 4	1.07 ± 0.07 ^bA^	0.94 ± 0.05 ^aA^
mean	0.9 ± 0.14 ^A^	0.84 ± 0.1 ^A^

^1^ Different lowercase letters indicate significant differences between harvest periods among samples of the same variety (*p* < 0.05). Different uppercase letters indicate significant differences between the two varieties at the same harvest period (*p* < 0.05).

**Table 5:** pH, TDS, CGA, caffeine and trigonelline contents of roasted coffee beans ^1^.

	7963	T8667
***pH***		
Harvest period 1	5.23 ± 0.01 ^aA^	5.22 ± 0.01 ^bA^
Harvest period 2	5.22 ± 0.01 ^aA^	5.21 ± 0.02 ^bA^
Harvest period 3	5.32 ± 0.02 ^bB^	5.14 ± 0.01 ^aA^
Harvest period 4	5.22 ± 0.02 ^aA^	5.31 ± 0.01 ^cB^
mean	5.25 ± 0.05 ^A^	5.22 ± 0.06 ^A^
***TDS* (%) **		
Harvest period 1	1.27 ± 0.13 ^aA^	1.37 ± 0.05 ^aA^
Harvest period 2	1.4 ± 0.12 ^abA^	1.35 ± 0.08 ^aA^
Harvest period 3	1.37 ± 0.06 ^abA^	1.45 ± 0.09 ^aA^
Harvest period 4	1.58 ± 0.16 ^cA^	1.4 ± 0.05 ^aA^
mean	1.41 ± 0.16 ^A^	1.39 ± 0.07 ^A^
***CGA (% d.b.)***		
Harvest period 1	1.21 ± 0.08 ^aA^	1.1 ± 0.06 ^aA^
Harvest period 2	1.22 ± 0.07 ^aA^	1.21 ± 0.06 ^aA^
Harvest period 3	1.36 ± 0.07 ^bA^	1.35 ± 0.09 ^bA^
Harvest period 4	1.67± 0.05 ^cB^	1.09 ± 0.08 ^aA^
mean	1.36 ± 0.2 ^A^	1.19 ± 0.13 ^A^
***Caffeine (% d.b.)***		
Harvest period 1	0.82 ± 0.06 ^bB^	0.75 ± 0.01 ^aA^
Harvest period 2	0.78 ± 0.002 ^aB^	0.76 ± 0.001 ^aA^
Harvest period 3	0.83 ± 0.01 ^bB^	0.79 ± 0.001 ^bA^
Harvest period 4	0.85 ± 0.01 ^cB^	0.81 ± 0.005 ^cA^
mean	0.82 ± 0.03 ^B^	0.78 ± 0.25 ^A^
***Trigonelline (% d.b.)***		
Harvest period 1	0.37 ± 0.015 ^aA^	0.39 ± 0.001 ^cA^
Harvest period 2	0.38 ± 0.001 ^aB^	0.35 ± 0.001 ^aA^
Harvest period 3	0.39 ± 0.06 ^bB^	0.37 ± 0.005 ^bA^
Harvest period 4	0.42 ± 0.004 ^cB^	0.35 ± 0.002 ^aA^
mean	0.39 ± 0.02 ^A^	0.37 ± 0.02 ^A^

^1^ Different lowercase letters indicate significant differences between harvest periods among samples of the same variety (*p* < 0.05). Different uppercase letters indicate significant differences between the two varieties at the same harvest period (*p* < 0.05).

## Data Availability

Data will be made available on request.
